# Characteristics of AgifCSP9 and Its Potential Involvement in Host Location by *Aphidius gifuensis* (Hymenoptera: Braconidae)

**DOI:** 10.3390/insects17100998

**Published:** 2026-09-27

**Authors:** Jun Jiang, Tao Huang, Huimin Qin, Tian Li, Xiaobin Zheng

**Affiliations:** 1 Dazhou Key Laboratory of Agricultural Resources Development and Ecological Conservation in Daba Mountain, Sichuan University of Arts and Science, Dazhou 635000, China; 20250033@sasu.edu.cn (J.J.); 18392362680@163.com (H.Q.); lt71200@163.com (T.L.); 2 Institute of Economic Crops, Dazhou Academy of Agricultural Sciences, Dazhou 635000, China; huang_tao2080@163.com

**Keywords:** *Aphidius gifuensis*, chemosensory protein 9 (CSP9), expression profiles, fluorescence competitive binding, molecular docking, pest management

## Abstract

Chemosensory proteins (CSPs) are a class of small soluble proteins traditionally considered to facilitate the recognition of chemical signals in insects. This study investigates the potential involvement of AgifCSP9 in host location and its response to host-related volatiles by RT-qPCR, ligand-binding assays, molecular docking, and dynamics simulations in *A. gifuensis*. Transcriptomic profiling revealed that *AgifCSP9* is highly expressed in the legs, with significantly higher relative expression levels in females and larvae compared to other developmental stages. Fluorescence competitive binding assays further demonstrated that AgifCSP9 exhibits relatively strong binding affinities to multiple host-related volatiles, among which sabinene, (Z)-β-ocimene, and myrcene displayed the highest affinities for AgifCSP9. Molecular docking and dynamics simulation analyses indicated that specific amino acid residues (Val9, Leu14, Ile40, Leu44, Ile67, and Phe70) of AgifCSP9 are involved in binding to these particular ligands. Collectively, these findings suggest that AgifCSP9 is a potential candidate molecule involved in host location by *A. gifuensis*, offering a promising molecular target for behavior-based pest management aimed at optimizing biological control efficiency.

## Simple Summary

*Aphidius gifuensis* (Hymenoptera: Braconidae) is a dominant endoparasitoid whose augmentative release has shown promise for managing aphid pests on various crops in China. Maximizing the biocontrol potential of *A. gifuensis* depends on deciphering its host recognition mechanisms, wherein chemosensory proteins (CSPs) serve as pivotal mediators of olfactory perception. In this study, we expressed AgifCSP9 in a prokaryotic system, evaluated its binding properties using fluorescence binding assays, and performed molecular docking and molecular dynamics simulations to validate its function. Our results demonstrate that AgifCSP9 binds three host-associated monoterpenoid compounds, providing a valuable reference for developing attractants to support natural enemy-based pest management.

## 1. Introduction

When attacked by herbivorous insects, plants may release volatile compounds to attract natural enemies, thereby defending themselves [1]. These natural enemies frequently rely on their olfactory system to locate and identify hosts by detecting host-related chemicals, such as herbivore-induced plant volatiles and pheromones. The process by which insects perceive these signaling cues is mediated by various olfactory-associated proteins [2,3]. Hydrophobic odor molecules enter the sensory lymph via cuticular pores on the antennal sensilla, where they bind to small proteins such as odorant-binding proteins (OBPs) or chemosensory proteins (CSPs). This complex is then transported to the dendritic membrane of olfactory sensory neurons (OSNs), where it activates odorant receptors, thereby inducing a transmembrane potential difference. The resulting membrane potential is transmitted to the central nervous system, ultimately eliciting a behavioral response [3,4,5].

In insects, CSPs are a family of highly conserved, small, water-soluble proteins characterized by a hydrophobic binding pocket nestled within α-helical domains [2]. While traditionally associated with chemo-sensation, CSPs exhibit remarkable functional versatility. Non-chemosensory roles of CSPs are well documented, encompassing embryonic development in *Apis mellifera* (AmelCSP5), oviposition in *Ophraella communa* (OcomCSP12), and insecticide resistance in *Plutella xylostella* (PxylCSP1) [6,7,8]. Additionally, CSPs facilitate visual pigment transport in *Helicoverpa armigera* [9] and mediate aphid-induced plant defenses via SmCSP4 [10]. Despite these diverse roles, the predominant function of CSPs in adult insects appears to be chemosensory, as most CSPs are highly expressed in the antennae and sensory organs of both pest insects and parasitoid wasps [11]. In parasitoids, CSPs are pivotal for perceiving both conspecific cues and host-associated volatiles. For instance, MmedCSP3 of the parasitoid wasp *Microplitis mediator* binds the noctuid sex pheromone components [12]. SnocCSP4 in *Sirex noctilio* specifically recognizes 10-oxodecanoic acid [13]. Concurrently, CSPs mediate the recognition of plant-derived semiochemicals, particularly herbivore-induced plant volatiles (HIPVs). AlepCSP2 in *Athetis lepigone* binds key green-leaf volatiles such as benzaldehyde and nonanal [14], and MmedCSP3 in *M. mediator* also exhibits high affinity for sesquiterpenes like (3E,7E)-4,8,12-trimethyltrideca-1,3,7,11-tetraene (TMTT) and 4,8-Dimethyl-1,3(E),7-nonatriene (DMNT) [12]. Nevertheless, despite these advances, the specific molecular mechanisms by which CSPs in parasitoid wasps mediate the recognition of host-plant volatiles remain poorly characterized.

*Aphidius gifuensis* (Hymenoptera: Aphidiidae) is a dominant endoparasitoid widely distributed across East Asia and the Hawaiian Islands, and within China, its range extends extensively from northern to southern provinces [15,16]. Numerous studies have confirmed *A. gifuensis* is a highly effective obligate parasitoid, exerting significant biological control over major aphid pests, including *Myzus persicae*, *Sitobion avenae*, and *Aphis gossypii* [15,17,18]. Based on the genome of *A. gifuensis*, we previously identified nine CSP genes [19]. Among these, AgifCSP5 was found to be involved in the recognition of the host plant volatile 2-nonanal by female wasps [19], potentially mediating oviposition behavior selection. However, other CSP members are known to exhibit diverse functional roles, suggesting that different CSPs may contribute to distinct aspects of chemosensory behavior. Highly expressed in legs, BodoCSP1 participates in attracting adult *Bradysia odoriphaga* to host-plant volatiles such as diallyl disulfide, dipropyl disulfide, and allyl methyl disulfide [20]. In light of this, and given our preliminary data showing high expression of *AgifCSP9* in the legs, the present study focuses exclusively on the functional characterization of *AgifCSP9* (Accession No.: XM_044157179). Firstly, we cloned *AgifCSP9* and determined its expression profiles across different developmental stages and tissues. Secondly, we characterized its binding affinities to host-related volatiles through fluorescence binding assays. Finally, we predicted protein binding sites through molecular docking and dynamics simulations. These findings suggest AgifCSP9 may be involved in the peripheral olfactory recognition of host volatiles and provide a basis for identifying candidate attractants for the biological control of aphids using *A. gifuensis*.

## 2. Materials and Methods

### 2.1. Plants and Insects

*M. persicae* was reared on cabbage (*Brassica oleracea* L. cv. ‘Jingfeng No. 1’). Both *M. persicae* and *A. gifuensis* were originally obtained from the Chongqing Key Laboratory of Entomology and Pest Control Engineering, College of Plant Protection, Southwest University, and subsequently maintained in our laboratory on cabbage hosts for more than five generations. Second- to third-instar nymphs of *M. persicae* were parasitized by *A. gifuensis*. Rearing conditions in the greenhouse were maintained at 25 ± 2 °C, a 14:10 h (L:D) photoperiod, and 30–70% humidity. Parasitized aphid mummies were individually collected and placed in Petri dishes (3.5 cm in diameter × 1 cm in height), the bottoms of which were lined with moistened filter paper, and humidity was maintained using soaked absorbent cotton balls. Following eclosion of adult parasitoid wasps, they were provided with a 15% glucose solution as a nutritional source.

### 2.2. Expression of AgifCSP9

Developmental-stage samples (20 fourth-instar larvae, 20 four-day-old pupae, and 30 adults per sex per group: mated at 1, 3, or 5 days) and dissected tissues of unmated 3-day-old adults (antennae, 80; heads without antennae, 40; thoraces, 40; abdomens, 40; legs, 80; wings, 80) were prepared. Total RNA was extracted using TRIzol reagent (TaKaRa, Dalian, China) following the manufacturer’s instructions. The quality and concentration of the extracted RNA were assessed using a NanoDrop 2000 spectrophotometer (Thermo Scientific, Wilmington, DE, USA). cDNA synthesis was carried out using the HiScript^®^ III All-in-one RT SuperMix Kit with gDNA Eraser Mix (Vazyme, Nanjing, China). The designed primers were used for RT-qPCR (Table S1). *β-actin* and *NADH* were selected as reference genes to normalize the expression levels of *AgifCSP9* across different developmental stages and tissues. RT-qPCR reactions were performed using the Taq Pro Universal SYBR qPCR Master Mix (Vazyme, Nanjing, China) on an ABI 7500 Real-Time PCR System (Applied Biosystems, Foster City, CA, USA). The reaction conditions were as follows: initial denaturation at 95 °C for 30 s, followed by 40 cycles of denaturation at 95 °C for 10 s and annealing at 60 °C for 30 s. Three biological replicates and three technical replicates were performed for each sample. The mRNA expression levels of *AgifCSP9* were calculated using the 2^−ΔΔCT^ method [21]. The data were first tested for normality and homogeneity of variance. One-way analysis of variance (ANOVA) was used to compare the relative expression levels of *AgifCSP9* among different developmental stages and between different tissues in females and males, with a significance threshold of *p* < 0.05. Statistical analyses were performed using SPSS version 26.0, and results are presented as mean ± standard error (SE).

### 2.3. Prokaryotic Expression and Purification of AgifCSP9

Primers containing *NdeI* and *EcoRI* sites were designed with Primer Premier 5.0 (Table S1) to amplify the *AgifCSP9* coding region. The PCR product was cloned into pTOPO-TA/Blunt vector (Aidlab, Beijing, China) and sequenced. After double digestion, the verified insert was subcloned into pET30a (+) and transformed into *E. coli* DH5α, then into BL21(DE3) for expression; at least five colonies were sequenced to confirm fidelity. Positive colonies were inoculated into 500 mL LB at 37 °C to optical density 600 (OD600) ≈ 0.6, induced with 1 mM IPTG for ≥6 h at 37 °C, and harvested (12,000× *g*, 20 min, 4 °C). Cells were resuspended, sonicated on ice, and centrifuged. The soluble supernatant was concentrated with a 3 kDa ultrafiltration tube (Millipore, Billerica, MA, USA; 5000 rpm, 4 °C, 45 min) and purified using ProteinIso^®^ Ni-NTA Resin (TransGen, Beijing, China), eluted with 300 mM imidazole in 50 mM NaH2PO4, 300 mM NaCl (pH 7.4). Purity was assessed by 15% SDS-PAGE and concentration by BCA kit (Solarbio, Beijing, China).

### 2.4. Fluorescence Binding Assays

To evaluate the binding affinity of AgifCSP9 to ligands, we used N-Phenyl-1-naphthylamine (1-NPN) as a fluorescent reporter and performed competitive fluorescence assays on a T2600 spectrofluorometer (Youke Instruments, Shanghai, China). Thirty-two volatile compounds were selected across three categories—aphid-derived semiochemicals, plant volatiles known to attract *A. gifuensis* or related parasitoids, and major tobacco leaf volatiles [22]—to evaluate binding selectivity comprehensively. The test set comprised alcohols ((+)-α-terpineol, cis-2-hexen-1-ol, cis-3-hexen-1-ol, leaf alcohol, cis-3-octen-1-ol), aldehydes (trans-2-hexen-1-al, cis-3-hexenal, hexadecanal, 1-pentanecarbaldehyde), alkenes ((-)-trans-caryophyllene, (E)-β-farnesene, myrcene, β-caryophyllene, (S)-(-)-limonene, (R)-(+)-limonene, (-)-α-pinene, 3-carene, sabinene, (Z)-β-ocimene, (1R)-(+)-α-pinene), and other compound classes ((R)-(-)-carvone, nepetalactone, cis-3-hexenyl acetate, methyl jasmonate, jasmonic acid, 6-methyl-5-hepten-2-one, acetosyringone, 1-pentanecarbaldehyde, methyl salicylate, hexadecane, eicosane, tridecane, nonadecane).

Each test compound was dissolved in chromatography-grade methanol to 100 mM, and diluted to 1 mM immediately before use. Evaluate ligand binding based on fluorescence intensity values, assuming that the protein is 100% active at saturation and that the stoichiometry is 1:1 (protein:ligand). The concentration of free ligand is calculated by subtracting the concentration of bound ligand from the total ligand concentration. AgifCSP9 was prepared at 2 μM in 20 mM Tris-HCl (pH 7.4). For 1-NPN binding, 2 μM protein was titrated with 1-NPN (2–20 μM). For competitive binding assays, increasing ligand concentrations (0–40 μM) were added to a fixed mixture of 2 μM AgifCSP9 and 2 μM 1-NPN. Fluorescence emission spectra were recorded from 350 to 500 nm with excitation at 337 nm (slit 10 nm), and the maximum fluorescence intensities from triplicate measurements were used to construct competition curves. The dissociation constant (Kd) for the AgifCSP9–1-NPN complex was calculated using Scatchard linear regression in GraphPad Prism 8.0 software (GraphPad Software Inc., La Jolla, CA, USA). Ligand binding affinity (Ki) was then calculated using the following equation:

Ki = [IC50]/(1 + [1 − NPN]/K1 − NPN)(1)

where IC50 is the ligand concentration displacing 50% of bound 1-NPN fluorescence, [1-NPN] is the concentration of free 1-NPN, and K1-NPN is the dissociation constant of the protein/1-NPN complex.

### 2.5. Three-Dimensional Structure and Molecular Docking of AgifCSP9

The three-dimensional model of AgifCSP9 was constructed using the online program AlphaFold3 (https://github.com/google-deepmind/alphafold3, accessed 13 April 2026) and the pTM score was 0.75. To evaluate the quality of the final three-dimensional model, the Structural Analysis and Verification Server (http://services.mbi.ucla.edu/SAVES/, accessed 14 April 2026), including Procheck and ERRAT, was employed. The three-dimensional structures of these ligands were downloaded from PubChem (https://pubchem.ncbi.nlm.nih.gov/, accessed 17 April 2026) in SDF format and subsequently converted to PDB format using Open Babel.

Protein–ligand complexes between AgifCSP9 and the selected compounds were constructed using Autodock Tools (v 1.5.6). After necessary preprocessing steps, including removal of water molecules, addition of hydrogen atoms, and charge calculation, the docking simulations between ligands and the receptor were performed using Autodock Vina (v 1.2.7). The conformation with the lowest binding energy was selected as the optimal binding mode for each complex. Visualization and analysis of the two-dimensional and three-dimensional structures of the proteins were carried out using Discovery Studio 2025 Client and Open-Source PyMOLTM (v 3.1.0, Schrödinger, LLC, New York, NY, USA).

### 2.6. Molecular Dynamics Simulation

Molecular dynamics (MD) simulations were performed using GROMACS 2022.2 [23]. Initial topology and coordinate files were generated via CHARMM-GUI [24]. The CHARMM36 force field [25] was applied to the protein, and ligand parameters were derived from CGenFF [26]. The protein–ligand complex was solvated in a 7 nm cubic box of TIP3P water [27] and neutralized with Na^+^/Cl^−^ ions. Energy minimization was performed using the steepest descent algorithm with position restraints applied to the protein backbone (400 kJ·mol^−1^·nm^−2^) and side chains (40 kJ·mol^−1^·nm^−2^) to relieve unfavorable contacts in the initial structure. The system was then equilibrated in two steps: first under NVT at 300 K (V-rescale, 10 ps), then under NPT at 1 bar (C-rescale isotropic, 50 ps), with a 2 fs timestep for both. Following energy minimization and equilibration, A 100 ns production simulation was subsequently carried out under NPT using Nose–Hoover temperature coupling and Parrinello–Rahman pressure coupling (2 fs timestep). Long-range electrostatic interactions were treated with the Particle Mesh Ewald (PME) method, while short-range non-bonded interactions were handled with a 1.2 nm cutoff. Bond lengths involving hydrogen atoms were constrained using the LINCS algorithm. From the resulting trajectories, root-mean-square deviation (RMSD) and root-mean-square fluctuation (RMSF) analyses were performed to evaluate the stability and conformational dynamics of the protein–ligand complexes. Data analysis and visualization were carried out using GROMACS utilities and the Lipid Nanobubble-Molecular Dynamics Toolkit (LNB-MDT) [28].

### 2.7. Binding Free Energy Calculation

Binding free energies were estimated using the MM/PBSA (molecular mechanics/Poisson–Boltzmann surface area) approach. From the equilibrated trajectory, 1000 evenly spaced frames (one per 100 ps) were extracted, and the binding free energy (ΔG_TOTAL) of each complex was computed with gmx_MMPBSA. The total binding free energy was calculated as:

ΔG_TOTAL = ΔE_VDWAALS + ΔE_EEL + ΔG_EPB + ΔG_ENPOLAR − TΔS(2)

with the entropic term (–TΔS) omitted in this analysis (thus reflecting only the enthalpic contribution). Mean binding energies, standard errors (SEM), and 95% confidence intervals (CI) were derived from statistics over the 1000 frames. Per-residue energy decomposition was performed using the same protocol, partitioning the total energy into side-chain and main-chain contributions for each residue, thereby identifying residues that favorably (negative values) or unfavorably (positive values) affect binding. All calculations were conducted with default parameters, and three independent simulation replicates were performed to ensure reproducibility.

## 3. Results

### 3.1. Expression Profile Analysis of AgifCSP9

The expression level of *AgifCSP9* was lowest on day 1 post-eclosion and subsequently increased progressively with time after emergence (Figure 1A; *df* = 23, *F* = 14.179, *p* < 0.001) in both females and males. Females exhibited significantly higher expression than males on days 1 and 5. Notably, larval expression surpassed that in pupae, males, and 1-day-old females, whereas pupae and 1-day-old males showed the lowest levels across all females and 3-day-old and 5-day-old males. Furthermore, *AgifCSP9* expression in the legs was significantly higher than that in the head, thorax, abdomen, antennae, and wings (Figure 1B; *df* = 17, *F* = 4.666, *p* = 0.001), with no differences observed between female and male tissues (Figure 1B).

### 3.2. Expression and Purification of AgifCSP9 Protein

The AgifCSP9 protein was successfully expressed in *E. coli* and purified from the supernatant, yielding a final concentration of 0.52 mg/mL. The presence of the His tag on the protein was confirmed by SDS-PAGE, which revealed a molecular weight of approximately 11 kDa, consistent with the predicted size (Figure 2A).

### 3.3. Binding Characteristics of AgifCSP9

The dissociation constant (Kd) of AgifCSP9 for 1-NPN was determined to be 2.34 μM (Figure 2B). 32 Different compounds were tested using competitive fluorescence binding assays (Table 1). Among these compounds, AgifCSP9 exhibited relatively higher binding affinities (Ki < 10 μM) for three test compounds, specifically sabinene (9.79 μM), (Z)-β-ocimene (9.34 μM), and myrcene (9.60 μM). AgifCSP9 showed relatively lower binding affinities (10 μM < Ki < 15 μM) for 15 compounds and weak or no binding affinities (Ki > 15 μM) for the remaining ligands (Figure 3).

### 3.4. Homology Modeling and Protein–Ligand Interactions

An ERRAT analysis showed that the overall quality factor of the model structure was 100% (Figure S1A), indicating that the non-bonded interactions among different atoms within the protein are reasonable. Furthermore, the Ramachandran plot revealed that 93.9% of the amino acid residues were located in the most favored regions (Figure S1B), demonstrating the reliability of the predicted model. Three-dimensional visualization analysis indicated that AgifCSP9 consists of five α-helices (α1–α5), located at residues Leu39-Leu43 (α1), Gln46-Leu57 (α2), Gly66-Gly79 (α3), Asp88-Thr101 (α4), and Glu106-Ala116 (α5) (Figure 4A). These structural features stabilize the internal cavity of the protein, which has a volume of 184 Å^3^ (Figure 4B).

Three high-affinity ligands with Ki values < 10 µM-(Z)-β-ocimene, myrcene, and sabinene -were selected from the fluorescence binding assays for further analysis. The molecular docking showed that the ligands bound to the hydrophobic pocket of AgifCSP9 with negative energy values, and the distances between potential interacting residues ranged from 3 Å to 6 Å (Table 2). Among these three compounds, (Z)-β-ocimene was able to form Pi-alkyl and alkyl hydrophobic interactions with AgifCSP9 through nine amino acid residues (Val9, Leu14, Ala17, Leu18, Phe23, Ile40, Leu44, Ile67, and Phe70), whereas the other two ligands formed only alkyl hydrophobic interactions with AgifCSP9 (Figure 5A). The binding of both myrcene and sabinene to AgifCSP9 relied primarily on alkyl hydrophobic interactions involving Val9, Leu14, Ile40, and Leu44 (Figure 5B,C).

### 3.5. Complex Stability

Both the AgifCSP9-(Z)-β-ocimene complex and its ligand exhibited large fluctuations after 40 ns, with peaks ranging from 4–9 Å, indicating instability in the binding environment and ligand binding (Figure 6A). The RMSD of the AgifCSP9-myrcene complex stabilized between 4–7 Å, whereas the small molecule showed large fluctuations after 60 ns, with peaks exceeding 10 Å, suggesting instability of the small-molecule binding (Figure 6C). Notably, the AgifCSP9-sabinene complex exhibited the smallest RMSD fluctuations: relatively large fluctuations were observed during the first 30 ns, after which the protein RMSD stabilized at 3–5 Å, while the ligand remained between 4–5 Å, indicating the best binding stability (Figure 6E). In the initial stage, the RMSD values of AgifCSP9 and sabinene fluctuated considerably. After 40 ns, both the protein and the ligands converged to approximately 3–5 Å. However, the ligands (Z)-β-ocimene and myrcene exhibited drastic fluctuations after 40 ns and 60 ns, respectively, indicating binding instability.

In all three complexes, the RMSF values of most AgifCSP9 residues ranged from 1–6 Å, with minimal conformational fluctuations in the core region. Only the N-terminal region (approximately the first six residues) showed a marked increase in RMSF (up to 7–12 Å), indicating high flexibility in this region. Different ligands exerted distinct effects on the residue flexibility of AgifCSP9: Binding to (Z)-β-ocimene resulted in the highest overall RMSF values, with higher flexibility in the core region and extreme flexibility at the N-terminus. Binding to sabinene produced the lowest RMSF values overall, but exhibited anomalous fluctuations at the C-terminus and a bimodal pattern of relatively high fluctuations in the binding pocket, indicating local instability. Binding to myrcene resulted in low flexibility overall, with the most gradual fluctuations at both the N- and C-termini (Figure 6B,D,F). The pronounced N-terminal and C-terminal fluctuations observed in the (Z)-β-ocimene complex correspond to the binding of multiple amino acid residues identified in the molecular docking results, such as Ala17, Leu18, Phe23 at the N-terminus and Ile67, Phe70 at the C-terminus.

### 3.6. Binding Free Energies and Key Residues

The total binding free energies of the three ligands with AgifCSP9 were −7.89 kcal/mol for (Z)-β-ocimene, −6.68 kcal/mol for myrcene, and −6.95 kcal/mol for sabinene (Figure 7). Since a lower binding free energy indicates stronger binding affinity, the order of affinity from strongest to weakest was (Z)-β-ocimene > sabinene > myrcene, although the differences among the three were not significant. Notably, the fluorescence competitive binding results also showed no significant differences in the binding affinities of the three compounds to AgifCSP9.

The binding of all three ligands to AgifCSP9 was dominated by van der Waals/hydrophobic interactions, whereas electrostatic and polar solvation contributions were not driving forces; in fact, polar solvation was the main opposing factor (Figure 7A,C,E). Despite accompanying substantial conformational fluctuations, myrcene achieves binding through a high degree of complementarity between its long hydrocarbon chain and the hydrophobic pocket. Sabinene, on the other hand, forms stable binding through precise interactions with core hydrophobic residues. (Z)-β-Ocimene not only matches the hydrophobic pocket via its long hydrocarbon chain but also relies on CH-π interactions, resulting in a more complex binding site architecture.

The core residues contributing to the binding of (Z)-β-ocimene to AgifCSP9, ranked by contribution strength, were Ile40 (−1.020 kcal/mol), Leu14 (−1.007 kcal/mol), Leu44 (−0.922 kcal/mol), Ile67 (−0.885 kcal/mol), and Phe70 (−0.807 kcal/mol). For the binding of myrcene to AgifCSP9, the core residues were Phe70 (−0.921 kcal/mol), Leu14 (−0.820 kcal/mol), Leu44 (−0.802 kcal/mol), Ile40 (−0.797 kcal/mol), and Ile67 (−0.706 kcal/mol). The core residues for sabinene binding to AgifCSP9 were Leu44 (−1.153 kcal/mol), Ile67 (−1.089 kcal/mol), Phe70 (−0.795 kcal/mol), Ile40 (−0.777 kcal/mol), and Leu14 (−0.709 kcal/mol) (Figure 7B,D,F).

## 4. Discussion

CSPs have been identified and characterized in various parasitoid wasp species, such as *Scleroderma guani*, *Cotesia vestalis*, *Cotesia ruficrus* and *M. mediator* [19,29,30,31]. In the study, *AgifCSP9* exhibited significantly higher expression in adult legs than in other tissues in *A. gifuensis*, a pattern consistent with *CSP9* in *Chilo suppressalis* [32] and *CSP1* in *B. odoriphaga* [20] implicated in host plant volatile recognition. Furthermore, the expression level of *AgifCSP9* was significantly higher in adults and larvae than in pupae. This expression pattern is consistent with that of *CSP3* in *Frankliniella occidentalis* [33], *Encarsia formosa* [34], and *Dendroctonus armandi* [35]. These three insect *CSP*s are known to be involved in the recognition of HIPVs. Intriguingly, on day 5 of adulthood, AgifCSP9 exhibited sex-biased expression, whereas no such difference was observed in the tissue expression profile. This discrepancy may be attributable to methodological differences: the tissue expression profiling used unmated wasps, whereas the developmental-stage analysis involved mated individuals. This suggests that mating status dynamically modulates *AgifCSP9* expression, potentially priming females for post-copulatory host search. Therefore, we speculate that the high expression of *AgifCSP9* in adults and larvae of *A. gifuensis* may be involved in host aphid location via HIPVs. Such upregulation post-mating aligns with observations in *C. ruficrus* and *Sesamia inferens* [36,37], and may explain why sex-based differences were obscured in tissue-specific assays. Notably, *AgifCSP9* showed female-biased expression, consistent with a function in perceiving host-derived signals like *CSP3* in *C. ruficrus* [36] during oviposition site location. In summary, AgifCSP9 likely functions as a transport carrier for volatiles in short-range communication that complements long-range olfaction, facilitating fine-scale assessment of host suitability during close-range inspection. Whether it harbors non-olfactory functions remains to be elucidated.

We confirmed that the N-terminal His tag did not compromise AgifCSP9 binding activity [9]. Subsequent binding assays showed that AgifCSP9 recognized 18 of 32 volatiles, suggesting its role in perceiving a wide range of plant volatiles. Notably, AgifCSP9 preferentially bound C10 non-polar monoterpenes (44% of active compounds) but showed negligible affinity for aphid cuticular volatiles or semiochemicals such as (E)-β-farnesene. These latter compounds are primarily detected by various olfactory proteins in other insects [38,39], reinforcing the functional specialization of AgifCSP9 for plant-derived monoterpenes. This observation also suggests that other CSPs or OBPs are likely required to bind polar or macromolecular compounds. In binding profiles, some compounds have clearly defined functions: crucifer-specific monoterpenes (limonene and myrcene) repel herbivores and attract *A. gifuensis* [40], while core HIPVs such as trans-2-hexenal, 1-pentanecarbaldehyde, and methyl salicylate also strongly attract the parasitoid [22]. In addition, (Z)-β-ocimene, myrcene, and sabinene are common monoterpenes constitutively released or induced by herbivory in many plants. Previous studies have shown that the emission of these compounds—especially ocimene and myrcene—can be significantly elevated upon aphid feeding, and they, as HIPVs [39,41], provide potential cues for natural enemies to locate host plants. Given that leg-enriched CSPs in other insects (e.g., *PxylCSP9* in *P. xylostella*) mediate host location via monoterpene recognition [40], we propose that AgifCSP9 similarly recognizes host-feeding-induced monoterpenes to locate its hosts. It may assist females in assessing habitat suitability at relatively close range by detecting plant-derived monoterpenes, thereby complementing the long-range olfactory search mediated by antennal highly expressed proteins. Beyond monoterpenoids, AgifCSP9 can also bind multiple other attractants, including trans-2-hexen-1-al, jasmonic acid, 1-pentanal, cis-3-hexenyl acetate, cis-3-hexen-1-ol, and methyl salicylate. However, RNA interference and behavioral assays are needed to confirm whether these candidate ligands trigger actual responses in *A. gifuensis*. Importantly, in the field, parasitoids such as *A. gifuensis* typically rely on complex blends of volatiles rather than single compounds for host location. The relative ratios, absolute concentrations, and temporal emission patterns of these three monoterpenes can convey specific information about host presence. Moreover, these common volatiles often act in combination with other HIPVs (e.g., green leaf volatiles, methyl salicylate) to create a characteristic odor signature that parasitoids can learn to associate with aphid-infested plants.

The three-dimensional structure of AgifCSP9 comprises five α-helices and four conserved cysteine residues that form a hydrophobic pocket, a configuration consistent with other insect chemosensory proteins [42,43]. Notably, AgifCSP9 contains one more α-helix than CrufCSP1, which possesses only four. This structural difference may result in a relatively smaller hydrophobic pocket, potentially favoring the specific recognition of certain odorant molecules and the formation of stable ligand–protein complexes [31]. However, the functional significance of this structural feature requires further experimental validation. Molecular docking identified nine residues—Val9, Leu14, Ala17, Leu18, Phe23, Ile40, Leu44, Ile67, and Phe70—within the hydrophobic pocket of AgifCSP9 as key residues mediating binding to (Z)-β-ocimene. The interactions are predominantly alkyl and π-alkyl hydrophobic contacts, a binding mode recognized as essential for odorant specificity. Similarly, myrcene and sabinene interact with AgifCSP9 primarily through alkyl hydrophobic contacts involving Val9, Leu14, Ile40, and Leu44. This interactive pattern is corroborated by analogous findings in other chemosensory proteins, including PxylCSP18 of *P. xylostella* with γ-terpinene [44], EforCSP3 of *E. formosa* with β-myrcene [34], and *BtabCSP2* of *Bemisia tabaci* with DMNT [45]. Among the three monoterpenes, (Z)-β-ocimene and myrcene are acyclic but differ in double-bond number and position—ocimene has three (including an internal (Z)-double bond), while myrcene has two with a terminal exo-methylene at C3—which alters their shape and flexibility, affecting pocket accommodation. Sabinene, however, is a bicyclic monoterpene with a rigid thujane skeleton and cyclopropane ring; its compact structure imposes steric hindrance that restricts conformational adaptability. Furthermore, AgifCSP9 exhibited the lowest docking energy among the tested ligands for the three monoterpenes, corroborating the results of the ligand binding assays. Collectively, these residues appear essential for conferring specificity toward monoterpenoid ligands, supporting the view that AgifCSP9 binds its ligands through defined recognition sites.

Molecular dynamics (MD) simulations revealed that sabinene, due to its rigid skeleton, is locked into a stable conformation, resulting in low protein–ligand RMSD, whereas the acyclic ligands, being more flexible, undergo continuous conformational adjustments within the pocket, leading to higher RMSD values without necessarily implying weaker binding affinities. This explains how minor structural differences lead to distinct binding behaviors. The key dynamic features of the AgifCSP9 binding pocket in N-terminus remained highly fluctuating even after ligand binding, whereas the flexible C-terminus appeared to undergo conformational adjustments that partially “envelop” the ligand. This encapsulation may shield the ligand from the aqueous environment, potentially enhancing binding stability [46,47]. The binding cavity was notably hydrophobic, consistent with our experimental observation that AgifCSP9 preferentially binds non-polar monoterpenes [48]. This hydrophobic micro-environment, shaped by these specific residues, likely enables AgifCSP9 to selectively filter and stably transport host-location cues—such as monoterpenes—from complex plant volatile blends, thereby assisting females in efficiently recognizing suitable habitats during close-range inspection. Nevertheless, while MD simulations offer a high-resolution view of protein–ligand dynamics, they cannot fully replicate the multifaceted experimental environment (e.g., variations in pH, ionic strength, and buffer composition), all of which can significantly modulate protein flexibility and ligand solubility [49]. Thus, whether AgifCSP9 indeed binds terpenoids through these residues warrants further validation via site-directed mutagenesis experiments.

## 5. Conclusions

In summary, our study demonstrates that AgifCSP9 exhibits relatively higher binding affinities to three host-related volatiles, implying that it may serve as a candidate molecule for host location. The results of molecular docking and molecular dynamics simulations underscore the importance of specific amino acid residues—namely Val9, Leu14, Ile40, Leu44, Ile67, and Phe70—in the binding of AgifCSP9 to these volatiles. These findings suggest that AgifCSP9 plays a potential role in host recognition. Understanding the olfactory targets of natural enemies such as AgifCSP9 is essential for developing effective pest management strategies. By identifying and characterizing the specific volatiles that interact with AgifCSP9, targeted approaches for pest control or enhancement of biological control efficacy can be devised. This knowledge contributes to the development of more efficient and environmentally sustainable strategies for crop protection and pest management.

## Figures and Tables

**Figure 1:**
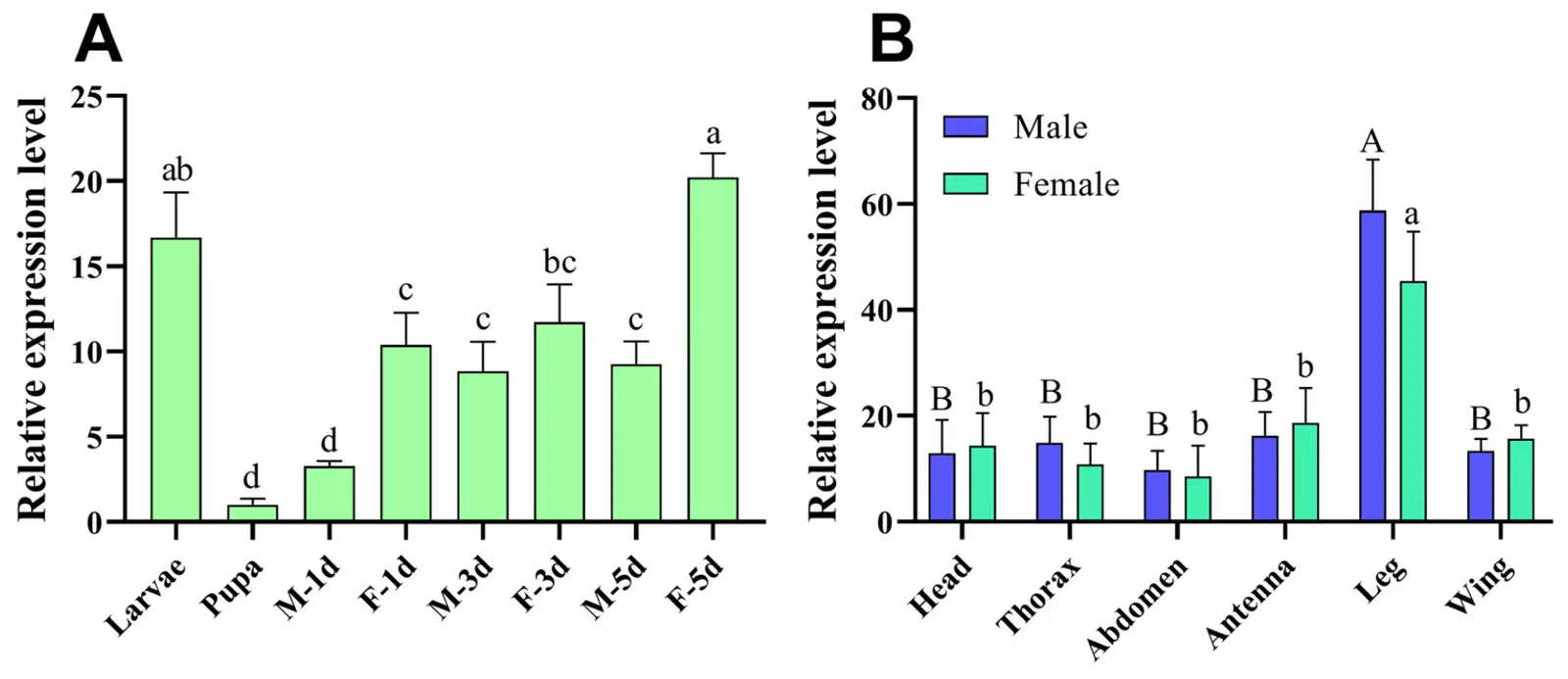
Expression levels (mean ± SE) of *AgifCSP9* at different developmental stages (**A**), and in various tissues (**B**) of *A. gifuensis*. Different uppercase/lowercase letters indicate significant differences between sexes and tissues (*p* < 0.05, One-way ANOVA). M: males; F: females.

**Figure 2:**
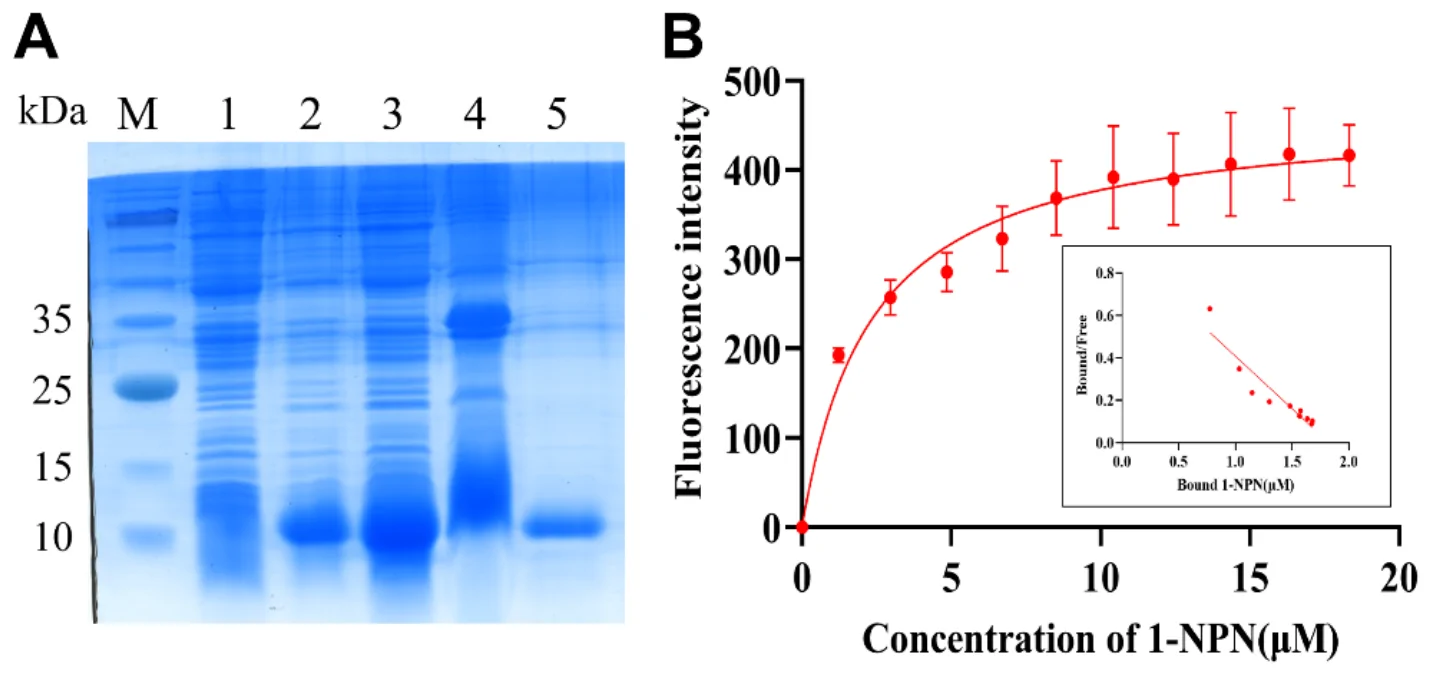
SDS-PAGE analysis of AgifCSP9 expression and purification (**A**), and 1-NPN binding curve with Scatchard plot (**B**). M: protein marker; lane 1: uninduced; lane 2: induced; lane 3: supernatant; lane 4: pellet; lane 5: purified His-tag AgifCSP9.

**Figure 3:**
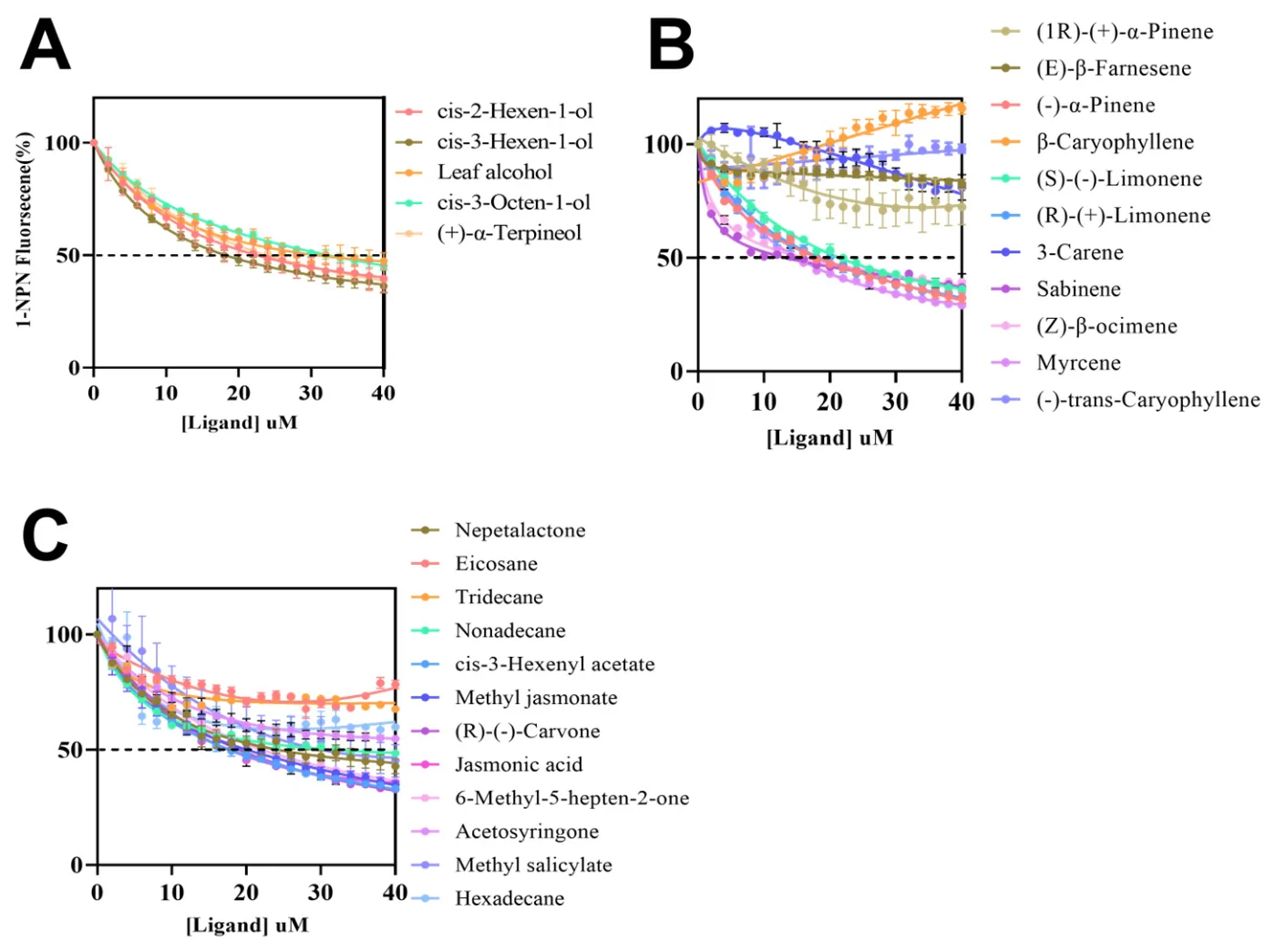
Binding curves of AgifCSP9 with alcohol (**A**), alkene (**B**), and other compounds (**C**).

**Figure 4:**
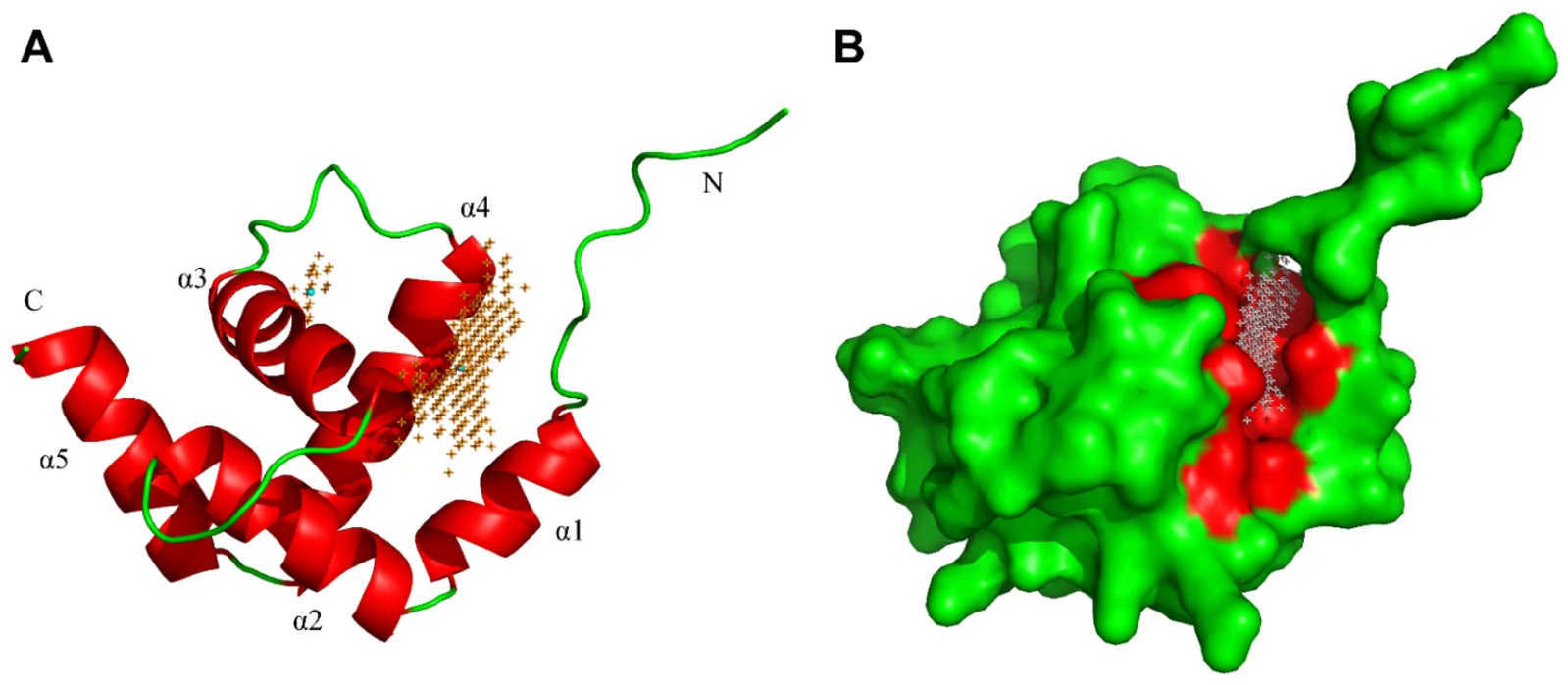
Three-dimensional structure of AgifCSP9 with predicted binding pocket highlighted in orange (**A**), and surface representation showing the pocket in silvery white (**B**). Light blue dots mark the pocket center. C and N labels denote the C-terminal and N-terminal regions, respectively.

**Figure 5:**
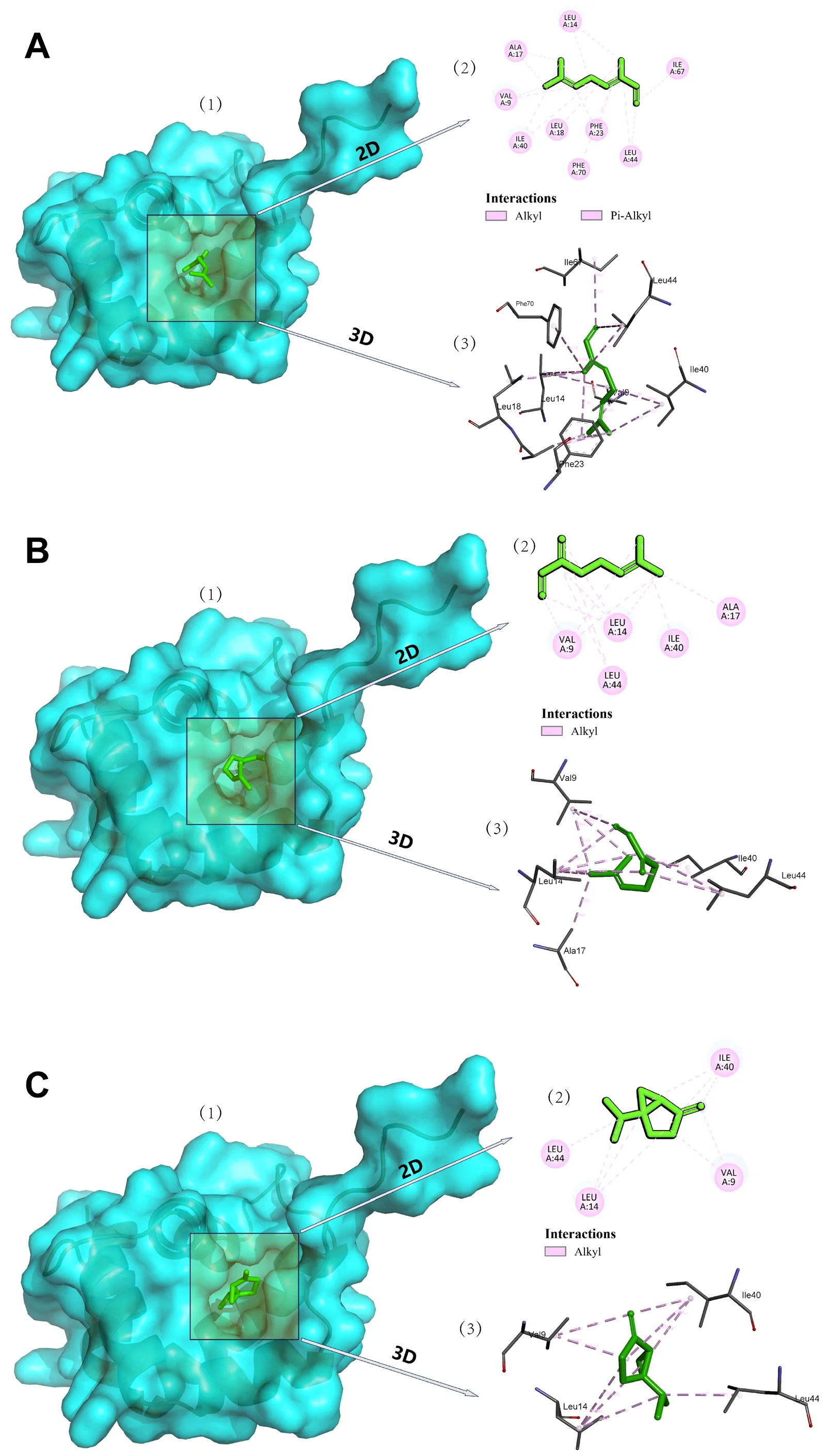
Molecular docking of AgifCSP9 with (Z)-β-ocimene (**A**), Myrcene (**B**), and Sabinene (**C**). In each panel, the panels show: showcases the optimal orientation and conformation of each ligand within the hydrophobic cavity formed by the hydrophobic residues of AgifCSP9 (1); displays the key residues of AgifCSP9 involved in the main interactions with the ligand in a 2D representation (2); and highlights the key residues of AgifCSP9 involved in the main interactions with the ligand in a 3D representation, and any Alkyl bonds formed are marked with pink dotted lines (3).

**Figure 6:**
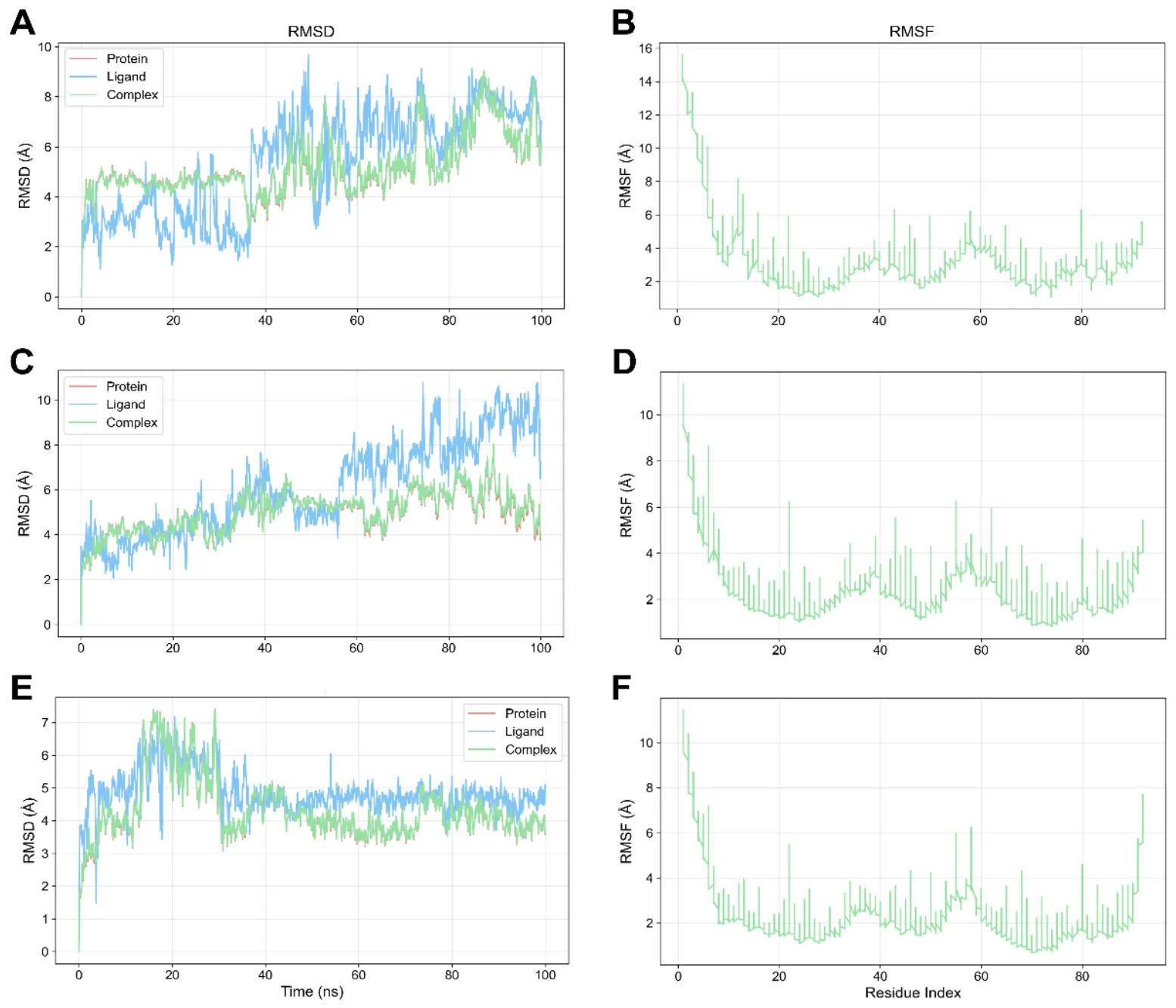
RMSD and RMSF analysis from molecular dynamics simulations of AgifCSP9 with (Z)-β-ocimene, Myrcene, and Sabinene. RMSD variation of (Z)-β-ocimene (**A**); RMSF of AgifCSP9 in the (Z)-β-ocimene system (**B**); RMSD variation in Myrcene (**C**); RMSF of AgifCSP9 in the Myrcene system (**D**); RMSD variation in Sabinene (**E**); RMSF of AgifCSP9 in the Sabinene system (**F**).

**Figure 7:**
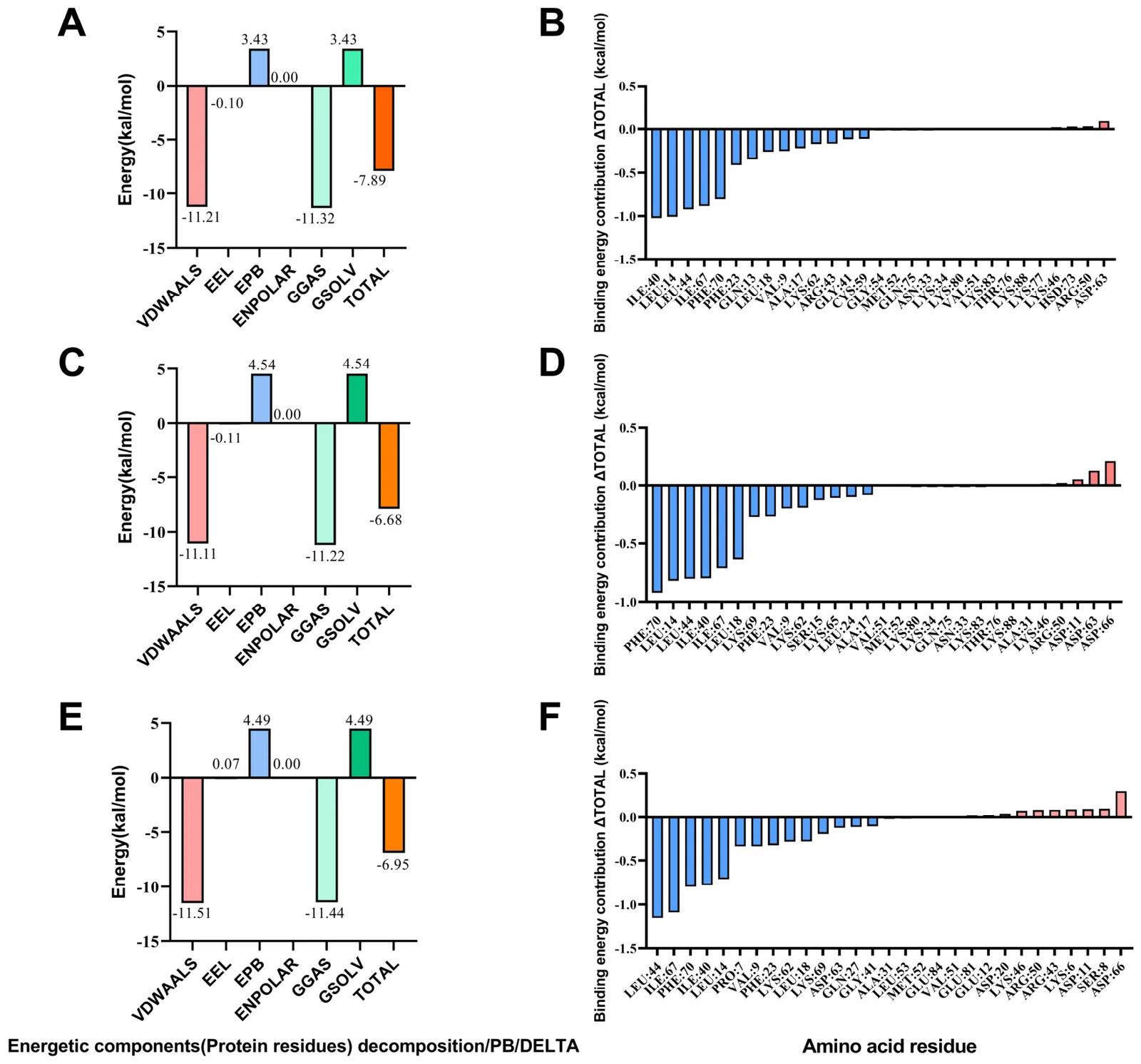
Energetic components decomposition (PB/DELTA) and amino acid residue binding energy contribution analysis of molecular dynamics simulations for AgifCSP9 with (Z)-β-ocimene, Myrcene, and Sabinene. Energetic components decomposition (PB/DELTA) for AgifCSP9 with (Z)-β-ocimene (**A**), Myrcene (**C**), and Sabinene (**E**), respectively. Corresponding residue contribution analyses with (Z)-β-ocimene (**B**), Myrcene (**D**), and Sabinene (**F**), respectively.

**Table 1:** Binding affinity of various ligands to AgifCSP9.

Ligand Name	CAS No.	Purity (%)	IC50	Ki (μM)
Aphid pheromone				
(1R)-(+)-α-Pinene	7785-70-8	97	17.92 ± 1.51	11.76 ± 0.99
Nepetalactone	21651-62-7	96	24.41 ± 8.68	16.02 ± 5.70
(E)-β-Farnesene	28973-97-9	80	>50	>50
(-)-α-Pinene	80-56-8	95	17.81 ± 0.59	11.68 ± 0.38
Eicosane	112-95-8	99	>50	>50
Tridecane	629-50-5	99	>50	>50
Nonadecane	629-92-5	99.7	32.56 ± 0.96	21.37 ± 0.63
Hexadecanal	629-80-1	97	22.05 ± 1.82	14.47 ± 1.20
Plant volatiles				
cis-2-Hexen-1-ol	928-94-9	95	23.87 ± 3.41	15.66 ± 2.24
β-Caryophyllene	329-53072	99	>50	>50
(S)-(-)-Limonene	5989-54-8	99	21.52 ± 2.10	14.12 ± 1.38
cis-3-Hexen-1-ol	928-96-1	99	18.42 ± 3.39	12.09 ± 2.23
cis-3-Hexenyl acetate	3681-71-8	95	17.36 ± 2.37	11.39 ± 1.55
(R)-(+)-Limonene	5989-27-5	95	19.76 ± 2.97	12.97 ± 1.95
trans-2-Hexen-1-al	6728-26-3	95	17.09 ± 0.77	11.21 ± 0.50
Methyl jasmonate	39924-52-2	95	20.06 ± 3.27	13.17 ± 2.14
(R)-(-)-Carvone	6485-40-1	98	19.31 ± 2.28	12.67 ± 1.50
cis-3-Hexenal	6789-80-6	90	22.47 ± 1.66	14.75 ± 1.09
Leaf alcohol	928-96-1	98	28.27 ± 6.70	24.98 ± 5.92
Jasmonic acid	77026-92-7	99	17.93 ± 1.00	11.76 ± 0.66
6-Methyl-5-hepten-2-one	110-93-0	99	22.81 ± 2.43	14.97 ± 1.60
Acetosyringone	2478-38-8	97	>50	>50
1-Pentanecarbaldehyde	66-25-1	98	17.58 ± 1.64	11.53 ± 1.08
Methyl salicylate	119-36-8	99	22.43 ± 7.78	14.72 ± 5.10
3-Carene	13466-78-9	90	>50	>50
Hexadecane	544-76-3	99	>50	>50
Sabinene	3387-41-5	75	14.91 ± 1.04	9.79 ± 0.68
(Z)-β-ocimene	13877-91-3	90	14.23 ± 1.12	9.34 ± 0.73
cis-3-Octen-1-ol	20125-84-2	98	32.94 ± 3.34	21.62 ± 2.19
(+)-α-Terpineol	7785-53-7	97	24.75 ± 2.97	16.24 ± 1.95
Myrcene	123-35-3	99.7	14.64 ± 0.99	9.60 ± 0.65
(-)-trans-Caryophyllene	87-44-5	98.5	>50	>50

**Table 2:** Docking of AgifCSP9 with three ligands.

Ligand	Binding Energy (kcal/mol)	Close-Contact Interacting Residues
(Z)-β-ocimene	−5.129	Val9 (3.91 Å), Leu14 (5.04 Å), Ala17 (3.60 Å), Leu18 (3.53 Å), Phe23 (4.44 Å), Ile40 (4.56 Å), Leu44 (4.51 Å), Ile67 (4.74 Å), Phe70 (5.16 Å)
Myrcene	−4.82	Val9 (4.05 Å), Leu14 (4.37 Å), Ala17 (3.60 Å), Ile40 (3.89 Å), Leu44 (4.67 Å)
Sabinene	−4.967	Val9 (4.13 Å), Leu14 (5.15 Å), Ile40 (3.91 Å), Leu44 (4.27 Å)

## Data Availability

Data are contained within the article or Supplementary Materials.

## Supplementary Materials

The following supporting information can be downloaded at: https://www.mdpi.com/article/10.3390/insects17100998/s1, Table S1: The primer sequences of AgifCSP9 for cloning and RT-qPCR. Figure S1A: ERRAT results of the constructed 3D model of AgifCSP9; Figure S1B: Ramanchandran plot of the constructed 3D model of AgifCSP9.
