# Supplementary material for: Characteristics of AgifCSP9 and Its Potential Involvement in Host Location by Aphidius gifuensis (Hymenoptera: Braconidae)
Source: Insects. 2026 Sep 27;17(10):998. doi: 10.3390/insects17100998 (PMC13705138; doi:10.3390/insects17100998)
Supplement: Supplementary file 1 [file insects-17-00998-s001.zip › insects-4541705-supplementary.pdf]

Table S1. The primer sequences of AgifCSP9 for cloning and RT-qPCR.

Application	Primer set	Forward (5′-3′)	Reverse (3′-5′)
Gene cloning	cAgifCSP9	GATGAGGTTACAGAAAAACCATCAG	TTATCCTGCCACAAATTTTTGTG
	qAgifCSP9	GTCCTTGTGATGCTATTG	CTAATATCTTTCAGGTCTTTACT
RT-qPCR	qActin	ACAGCAGCATCATCATCAA	TCTGGACAACGGAATCTTT
	qNADH	CTGGCACTGGGATAAAAC	TCAGGAATTGGTGAAAGC
Vector	vAgifCSP9	CC<u>CATATG</u>	CG<u>GAATTC</u>
construction		GATGAGGTTACAGAAAAACCATCAG	TTATCCTGCCACAAATTTTTGTG

Note: Underlining indicates the sequence of restriction sites.

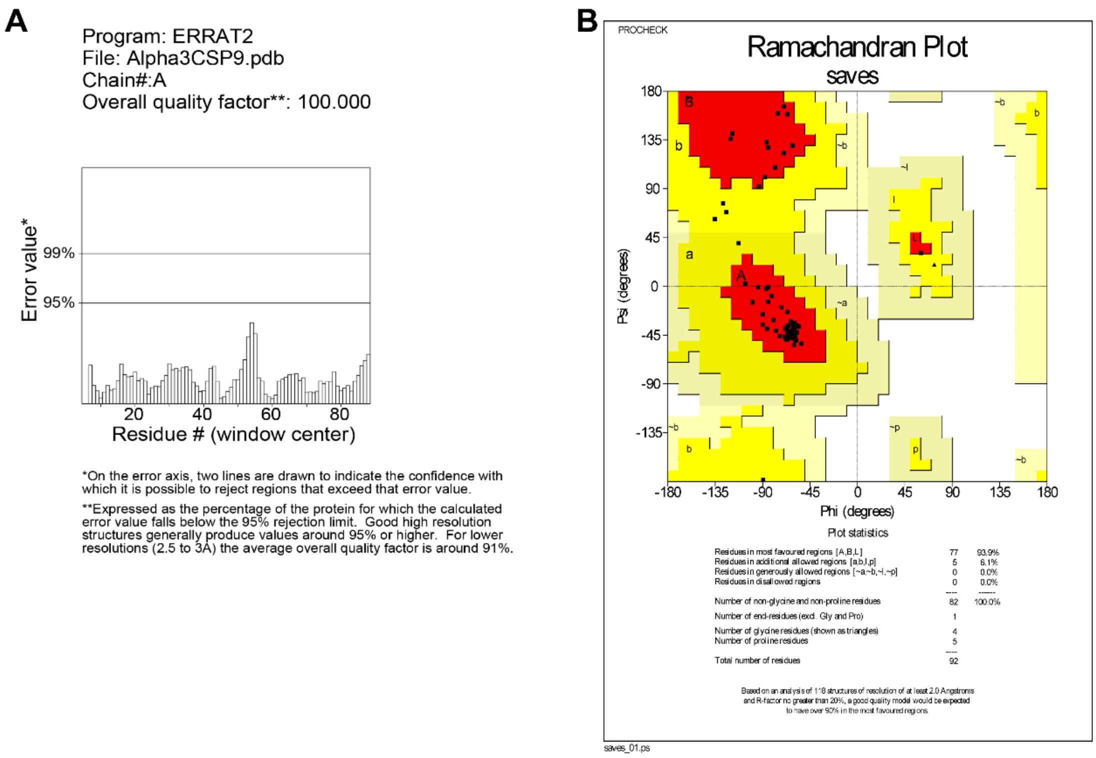

Figure S1. (A): ERRAT results of the constructed 3D model of AgifCSP9; **(B):** Ramanchandran plot of the constructed 3D model of AgifCSP9.
